# The risk of childhood autism among second-generation migrants in Finland: a case–control study

**DOI:** 10.1186/1471-2431-13-171

**Published:** 2013-10-19

**Authors:** Venla Lehti, Susanna Hinkka-Yli-Salomäki, Keely Cheslack-Postava, Mika Gissler, Alan S Brown, Andre Sourander

**Affiliations:** 1 Department of Child Psychiatry, University of Turku, Lemminkäisenkatu 3 / Teutori, Turku 20014, Finland; 2 Department of Epidemiology, Mailman School of Public Health, Columbia University, Allan Rosenfield Building, 722 West 168th Street, New York, NY 10032, USA; 3 National Institute of Health and Welfare (THL), P.O. Box 30, Helsinki 00271, Finland; 4 Nordic School of Public Health, P.O. Box 12133, Gothenburg SE 40242, Sweden; 5 New York State Psychiatric Institute, Department of Psychiatry, College of Physicians and Surgeons of Columbia University, 1051 Riverside Drive, Unit 23, New York, NY, USA

**Keywords:** Autism, Risk factor, Parental, Migration, Epidemiology

## Abstract

**Background:**

Studying second-generation immigrants can help in identifying genetic or environmental risk factors for childhood autism. Most previous studies have focused on maternal region of birth and showed inconsistent results. No previous study has been conducted in Finland.

**Methods:**

The study was a nested case–control study based on a national birth cohort. Children born in 1987–2005 and diagnosed with childhood autism by the year 2007 were identified from the Finnish Hospital Discharge Register. Controls were selected from the Finnish Medical Birth Register. Information on maternal and paternal country of birth and mother tongue was collected from the Finnish Central Population Register. There were 1132 cases and 4515 matched controls. The statistical test used was conditional logistic regression analysis.

**Results:**

Compared with children with two Finnish parents, the risk of childhood autism was increased for those whose parents are both immigrants (adjusted odds ratio [aOR] 1.8, 95% confidence interval [CI] 1.2–2.7) and for those with only an immigrant mother (aOR 1.8, 95% CI 1.2–2.7), but not for those with only an immigrant father. The risk was increased for those with a mother born in the former Soviet Union or Yugoslavia and for those with a mother or a father born in Asia. Specific parental countries of birth associated with an increased risk were the former Soviet Union, the former Yugoslavia and Vietnam.

**Conclusions:**

In Finland, children who are born to immigrant mothers with or without an immigrant partner, have an increased risk of childhood autism. The risk varies with immigrant parents’ region of birth. The findings may help in identifying possible risk factors, which can be examined in future studies.

## Background

Autism spectrum disorders (ASD) are neurodevelopmental disorders characterized by impaired social interaction and communication and by restricted, stereotyped and repetitive patterns of behavior. Childhood autism is the most severe form with the poorest outcome. Its etiology is largely unknown. There is strong evidence of genetic contribution including both inherited factors and de novo mutations [1,2]. Heritability rate as high as over 90% has been suggested for childhood autism [3]. This indicates that most of the variation in risk for childhood autism in the studied populations can be explained by genetic factors, but it does not, however, give information on the genetic contribution to individual’s phenotype [4]. Environmental factors, possibly through gene-environment interactions and correlations influence the risk of autism as well [2,5,6]. Examples of potential environmental risk factors are advanced parental age, obstetric complications, dietary factors, lack of vitamin D, and different mutagenic chemicals [5,7,8], which may also reflect underlying genetic effects and interaction or correlation with genetic factors. Second-generation immigrants are an important group for studying the etiology of childhood autism, because parents from different regions may differ in terms of genetic risk, but they may also have been exposed to different levels of environmental risk factors before or after immigration.

Many previous studies conducted in Europe have shown that immigrant mothers born outside Europe have an increased risk of having a child with childhood autism [9-11] or other ASD [12], but there are also studies reporting a non-significant association [13,14]. In one study maternal immigration was associated only with ASD with intellectual disability [15]. Outside Europe, an Australian study showed that immigrant mothers had an increased risk of having a child with ASD [16]. A California study showed no increased risk for any immigrant mothers, although Mexican-born mothers had a decreased risk of having a child with childhood autism [17]. The studies on paternal country of birth have been conducted in Sweden and Denmark and their findings have been inconsistent. Two studies have shown an association between father’s, but not mother’s immigrant background, and childhood autism [13] or ASD [14] in offspring. One study has shown an association between mother’s, but not father’s immigrant background, and childhood autism [18]. It has also been shown that having only an immigrant mother [10,11] or two immigrant parents [10] is associated with childhood autism, but having only an immigrant father is not.

The comparison of previous studies is complicated by small samples and variation in the immigration profiles of different countries. In addition, only a few such studies [9,11,13,18] have been nationally representative. In several studies, subjects and information on covariates have been collected from national or statewide registers [9,11,13,15,18], while in two studies the cases have been obtained from clinics in a certain region, but controls and information on covariates have been collected from a national or statewide register [10,16]. In one study the cases were ascertained from only a few clinics, and a population comparison group comprised only census data [12]. There has also been variation in the definition of outcomes. In most studies the outcome has been either childhood autism or the entire ASD spectrum.

Finland is a country with a relatively low, but constantly increasing number of immigrants. In 1990 the proportion of foreign-born people in the Finnish population was only 1.3% while in 2010 it was 4.8% [19]. In 2010 the most common categories under which residence permits were issued included working, studying and family relations, accounting for 70% of all immigrants [20,21]. Immigrants generally use both primary and specialized health services less than Finnish people and it has been estimated that they may also be healthier, but there is great variation within the immigrant population [22]. This case–control study is based on a large national cohort, and the use of comprehensive register data provides information on all children diagnosed with childhood autism in specialized health care and a representative sample of controls. The aim of this study was to examine the associations of maternal and paternal region of birth with childhood autism in offspring. Based on previous European studies our hypothesis was that the offspring of non-European parents would have an increased risk of childhood autism. In particular, we hypothesized that offspring of mothers with dark skin would have the highest risk, based on a suggested association between lower levels of vitamin D and increased risk of autism in offspring [8,23-25].

## Methods

The study is derived from the Finnish Prenatal Study of Autism (FIPS-A), which is a nested case–control study based on a national birth cohort, and aims to identify early life risk factors of ASD. The methods have been described in detail by Lampi et al. [26]. The study was authorized by the Ministry of Social Affairs and Health of Finland (STM/2593/2008) with approvals from the National Institute for Health and Welfare (THL), the Ethics Committee of the Intermunicipal Hospital District of Southwest Finland, and the Institutional Review Board of the New York State Psychiatric Institute. To assess the association between parental region of birth and childhood autism, we conducted a linkage between three national registers for 1132 cases and their 4515 controls born in 1987–2005 and matched by age, sex and region.

### Case and control identification

Children born in 1987–2005 and diagnosed with childhood autism by the year 2007 were identified from the Finnish Hospital Discharge Register (FHDR), a nationwide register maintained by THL. It includes the personal identification numbers and covers the days of admission and discharge in all public and private inpatient care units in Finland for the whole follow-up period and the outpatient visits in hospitals since 1998. The diagnoses included in the register are based on the International Classification of Diseases (ICD). In this study the diagnostic code 299.0 in ICD-9 (years 1987–1995) and F84.0 in ICD-10 (years 1996–2007) were used. A validation study has shown that the validity of childhood autism diagnosis in the FHDR is very good [27].

Four controls per case were selected from the Finnish Medical Birth Register (FMBR), which is another mandatory national register maintained by THL. It includes information on maternal background, pregnancy, and the prenatal and neonatal period up to age seven days on all births in Finland. The register includes mothers’ personal identification numbers linked to children. The controls were matched to each case by date of birth (+/- 30 days), region of birth, sex, and residence in Finland. The exclusion criteria for controls were ASD or severe/profound mental retardation according to the FHDR. Of the originally matched 4528 controls, 12 children and ten mothers had invalid or incomplete personal identification numbers. This led to a removal of 13 controls from the case–control database leading to 4515 controls, since no follow-up data could be gathered for them.

### Parental immigration status

The data on parental country of birth and mother tongue were collected from the Finnish Central Population Register (CPR), which is a computerized national register that contains basic information about Finnish citizens and foreign citizens residing permanently in Finland. Asylum seekers and recent migrants without personal identification numbers are not included in the register. The register includes personal identification numbers which are issued to all Finnish citizens and permanent residents at birth or at migration. Parents can be identified by linking their personal identification number with that of their children’s. In this study the focus was on parents who are first-generation immigrants. They were defined as those who were born abroad and whose mother tongue is not Finnish. Those who were born in Finland and/or whose mother tongue is Finnish were defined as Finnish.

Three different methods were used for classifying parents. First, a four-category variable was used for the primary analysis: both parents Finnish (reference), mother immigrant and father Finnish, father immigrant and mother Finnish and both parents immigrants. Second, a regional analysis was conducted separately for mothers and fathers using the following categorization: 1) Finnish (reference), 2) Western countries (most European countries, North America, Australia and New Zealand), 3) Countries which were part of the Soviet Union or Yugoslavia, 4) Sub-Saharan Africa, 5) North Africa and Middle East, and 6) Asia (excluding Middle East). Both geographical and socioeconomic factors were considered for the categorization. All “Western countries” are members of OECD (Organization for Economic Co-operation and Development) except for Romania and Bulgaria, which are nevertheless members of the European Union. Latin American countries were excluded from this analysis, because the group was very small with only three controls and four cases. Third, a country-specific analysis was conducted separately for mothers and fathers using countries from which there were at least ten mothers or fathers in the sample. Being Finnish was used as a reference.

### Covariates

The inclusion of covariates was based on analyses of bivariate associations between: 1) selected variables from the FMBR or CPR and childhood autism, and 2) these same variables and immigration status among controls. The results of these analyses are shown in Table 1. Since only paternal age and maternal age were significantly associated with both exposure and outcome, these two variables were included as covariates in adjusted models. Parental age was considered to be a possible confounder. Table 1 shows that the age of immigrant parents differs from Finnish parents especially in families with both an immigrant mother and father. Advanced parental age as a risk factor for childhood autism has been described in more detail in a previous study based on FIPS-A [28]. Parents’ socioeconomic status (SES) was not included as a possible confounder, since the variable available in FMBR is considered to be unreliable in refugee populations with incomplete information on their education and many of them are outside the labour force, e.g. in education or at home.

**Table 1:** Covariates in relation to immigration status in controls and in relation to the risk of childhood autism

	**Immigration**					**Relationship between covariates and childhood autism p-value**^**a**^
**Covariates**	**Both parents Finnish n (%)**	**Mother only immigrated n (%)**	**Father only immigrated n (%)**	**Both parents immigrated n (%)**	**p-value**^**a**^	
Maternal age (≥median, 29 years)	2 386 (55.9)	48 (60.0)	48 (62.3)	34 (36.6)	0.001	<0.001
mean (years)	29.5	30.3	30.1	27.4		
SD (years)	5.3	5.2	5.2	4.7		
Paternal age (≥median, 32 years)	2 055 (48.2)	47 (58.8)	41 (53.3)	58 (62.4)	0.01	0.003
mean (years)	31.9	35.7	32.6	33.0		
SD (years)	5.9	7.7	6.7	5.8		
Smoking^b^	714 (17.2)	10 (13.2)	10 (13.2)	1 (1.2)	<0.001	0.77
Previous births (≥2)	1 105 (25.9)	15 (18.8)	18 (23.4)	36 (38.7)	0.02	0.22
Pre-term birth (<37 weeks)	229 (5.4)	8 (10.0)	1 (1.3)	7 (7.5)	0.08	0.06
Low birthweight (<2500 g)	135 (3.2)	3 (3.8)	0 (0)	3 (3.2)	0.45	<0.001
mean (g)	3598	3558	3580	3539		
SD (g)	546	450	490	529		

^a^X^2^ test, ^b^frequency missing = 47 cases, 132 controls. OR=odds ratio, CI=confidence interval.

### Statistical analysis

The analysis was based on a nested case–control design, where the controls for each case were matched from the population at risk on selected factors, elaborated in “Case and control identification”. To analyze the primary outcome, the four-level variable describing parents’ immigration status was utilized. To study the regional associations, we utilized immigrant parents’ country of birth. They were categorized in the five geographic regions described above, and elaborated in “Parental immigration status”. The reference group in each analysis was “Finnish parents”. Point and interval estimates of odds ratios were obtained by fitting conditional logistic regression models for matched sets. A p-value of less than 0.05 was considered statistically significant. Statistical analyses were performed with SAS software (SAS 9.2, SAS Institute, Cary, NC, USA).

## Results

Among all children with childhood autism, 8.6% had at least one immigrant parent. Among controls, 5.5% had an immigrant parent. Cases with two immigrant parents had been diagnosed with childhood autism at significantly younger age than cases with two Finnish parents. The average age at diagnosis was 3.8 years for those with two immigrant parents and 5.6 years for those with two Finnish parents (p=0.002). Those with one immigrant parent did not significantly differ from those who have two Finnish parents. Compared with children in whom parents were both Finnish, the risk of childhood autism was increased for those whose parents were both immigrants (adjusted OR 1.8, 95% CI 1.2–2.7) and for those with only an immigrant mother (1.8, 1.2–2.7), but not for those with only an immigrant father (Table 2).

**Table 2:** Immigration status by childhood autism in cases and controls

	**Cases n (%)**	**Controls n (%)**	**OR (95% CI)**	**p**	**Adjusted**^**a**^**OR (95% CI)**	**p**
**Both parents Finnish**	1 035 (91.4)	4 265 (94.5)	Ref.		Ref.	
**Mother only immigrated**	35 (3.1)	80 (1.8)	1.8 (1.2-2.7)	0.004	1.8 (1.2-2.7)	0.004
**Father only immigrated**	23 (2.0)	77 (1.7)	1.2 (0.8-2.0)	0.39	1.3 (0.8-2.1)	0.34
**Both parents immigrated**	39 (3.5)	93 (2.1)	1.8 (1.2-2.6)	0.004	1.8 (1.2-2.7)	0.002

^a^Adjusted for parental age**.** OR=odds ratio, CI=confidence interval.

The regional analysis was conducted separately for maternal and paternal region of birth. Significant associations were observed between region of birth and childhood autism in offspring (Table 3). The risk of childhood autism was increased for those with mothers (adjusted OR 1.8, 95% CI 1.2–2.9) born in the former Soviet Union or Yugoslavia and for those with mothers (2.6, 1.4–4.7) or fathers (4.4, 2.0–9.5) born in Asia.

**Table 3:** Maternal and paternal region of birth by childhood autism in cases and controls

	**Cases**		**Controls**		**OR (95% CI)**	**p**	**Adjusted**^**a**^**OR (95% CI)**	**p**
	**n**	**%**	**n**	**%**				
**Mothers**								
Finnish	1 058	93.9	4 340	96.2	Ref.		Ref.	
Western countries	10	0.9	29	0.6	1.4 (0.7-2.9)	0.36	1.4 (0.7-2.9)	0.37
Former Soviet Union and former Yugoslavia	29	2.6	65	1.4	1.8 (1.2-2.8)	0.008	1.8 (1.2-2.9)	0.007
Sub-Saharan Africa	10	0.9	36	0.8	1.2 (0.6-2.4)	0.68	1.2 (0.6-2.6)	0.57
North Africa, Middle East	2	0.2	14	0.3	0.6 (0.1-2.6)	0.49	0.6 (0.1-2.7)	0.50
Asia	18	1.6	28	0.6	2.6 (1.4-4.7)	0.002	2.6 (1.4-4.7)	0.002
**Fathers**								
Finnish	1 069	94.9	4 344	96.3	Ref.		Ref.	
Western countries	8	0.7	43	1.0	0.7 (0.4-1.6)	0.46	0.7 (0.4-1.6)	0.45
Former Soviet Union and former Yugoslavia	15	1.3	35	0.8	1.7 (0.95-3.2)	0.07	1.8 (0.97-3.3)	0.06
Sub-Saharan Africa	13	1.2	38	0.8	1.4 (0.7-2.8)	0.29	1.5 (0.8-3.0)	0.21
North Africa, Middle East	9	0.8	38	0.8	1.0 (0.5-2.0)	0.90	1.0 (0.5-2.1)	0.98
Asia	13	1.2	12	0.3	4.4 (2.0-9.6)	<0.001	4.4 (2.0-9.5)	<0.001

^a^Adjusted for parental age**.** OR=odds ratio, CI=confidence interval.

In the country-specific analysis, which as noted above, was conducted for countries from which there were at least ten mothers or fathers, the following countries were included: the former Yugoslavia, the former Soviet Union, Turkey, Thailand, Vietnam and Somalia. Birth of a parent in Vietnam or Yugoslavia, or birth of a mother in the former Soviet Union were significantly associated with childhood autism in offspring. Parental birth in other countries was not associated with autism. The results are shown in Table 4.

**Table 4:** Maternal and paternal country of birth by childhood autism in cases and controls

	**Cases**		**Controls**		**OR (95% CI)**	**p**	**Adjusted**^**a**^**OR (95% CI)**	**p**
	**n**	**%**	**n**	**%**				
**Mothers**								
Finnish	1 058	93.9	4 340	96.2	Ref.		Ref.	
Former Yugoslavia	6	0.5	8	0.2	3.0 (1.1-8.8)	0.04	3.2 (1.1-9.1)	0.03
Former Soviet Union	22	2.0	50	1.1	1.7 (1.05-2.9)	0.03	1.7 (1.05-2.9)	0.03
Somalia	6	0.5	34	0.8	0.7 (0.3-1.8)	0.49	0.8 (0.3-1.9)	0.60
Thailand	3	0.3	13	0.3	1.0 (0.3-3.6)	0.9885	1.0 (0.3-3.7)	0.95
Vietnam	5	0.8	9	0.1	7.0 (2.3-20.9)	<0.001	7.0 (2.3-21.2)	<0.001
**Fathers**								
Finnish	1 069	94.9	4 344	96.3	Ref.		Ref.	
Former Yugoslavia	8	0.7	8	0.2	4.0 (1.5-10.6)	0.006	4.1 (1.5-10.9)	0.005
Former Soviet Union	5	0.5	21	0.5	0.9 (0.4-2.5)	0.88	0.9 (0.4-2.5)	0.89
Somalia	8	0.7	31	0.7	1.1 (0.5-2.6)	0.78	1.2 (0.5-2.8)	0.62
Turkey	1	0.1	10	0.2	0.4 (0.1-3.3)	0.40	0.5 (0.1-3.5)	0.45
Vietnam	8	0.7	5	0.1	6.4 (2.1-19.7)	0.001	6.4 (2.1-19.5)	0.001

^a^Adjusted for parental age. OR=odds ratio, CI=confidence interval. Countries with ten or more immigrant mothers or fathers were included.

## Discussion

This study showed that in Finland, children have an increased risk of childhood autism if their mother or both parents are immigrants. This is in line with the two previous studies which have used a similar categorization as the present study [10,11]. The fact that having only an immigrant father did not significantly increase the risk suggests that risk factors specific to the mother, such as prenatal adversity, may play a role. It is also possible, however, that immigrant fathers in families with a Finnish mother differ from other immigrant fathers by their region of origin or by other factors. The regional analysis showed that both maternal and paternal birth in Asia or maternal birth in the former Soviet Union/Yugoslavia increased the offspring’s risk of childhood autism while the risk was not increased for children whose parents were born in any of the other regions.

Our hypothesis about an increased risk of childhood autism in non-European parents was only partially supported. The findings did not suggest an explanation related to socioeconomic factors. In Africa and the Middle East there are many low-income areas from which Finland receives asylum seekers and refugees, but the risk of autism among immigrants from these regions did not differ substantially from those born in high-income regions. In addition, even though there was no information on parents’ ethnicity or skin color, this does not appear to present a coherent explanation for the regions with increased risk. Having a parent from a region with predominantly dark-skinned people such as Sub-Saharan Africa was not associated with an increased risk of childhood autism. This does not support our second hypothesis of dark-skinned parents having a particularly high risk of childhood autism in offspring. A British study suggested that immigration itself instead of ethnicity is the primary risk factor [12]. Previously it has been suggested that a possible explanation for the increased risk of autism among children whose parents have immigrated from Southern to Northern latitudes shown in some studies could be alterations in the immune repertoire due to differences in early pathogen exposure in mothers or neonates [29]. In this study there was no clear South–North difference, but this does not exclude the possibility of immunological factors being part of the mechanism. The role of immune system in autism has also been supported by the findings of an earlier study on this cohort [30].

Two previous studies have also shown an increased risk of childhood autism among children who have a mother born in Asia [10,16]. Two other studies have not shown an association between maternal birth in Asia and ASD in offspring [14,15], but one of them showed an increased risk associated with an Asian father [14]. There are no previous studies with a focus on the former Soviet Union or Yugoslavia. Furthermore, no prevalence studies on autism have been conducted in the former Soviet Union and Yugoslavia and few studies have been conducted in Asia. A review showed that most Asian studies have been conducted in Japan where the prevalence estimates of ASD have ranged between 0.02–1.8%, in line with studies from other regions [31]. In a Korean sample the prevalence of ASD was found to be as high as 2.6%, but the study differed from most previous studies in using rigorous screening of a large community [32]. Hence, it is not known if the increased risk detected among children of immigrants from Asia or the former Soviet Union and Yugoslavia who live in Finland would be found in their parents’ countries of origin as well.

The former Soviet Union is a very large region and immigrants from there comprise such a heterogeneous group that it is very difficult to develop hypotheses on specific factors that may explain the association. The former Yugoslavia and Vietnam, however, are smaller and immigrants from those countries may form more homogeneous groups. Interestingly, both countries were afflicted by war, and many immigrants are likely to be refugees. The number of people born in Vietnam has increased in Finland, from 1550 in 1990 to 4490 in 2010 [19]. In 1990 there were about 140 people born in the former Yugoslavia, but 8000 in 2010 [19]. In this sample 93% of children with a Vietnamese parent and 76% with a Yugoslavian parent had both parents born in the same country. While the increased risk associated with fathers from these regions may be mediated by maternal factors, it is also possible that there are genetic risk factors or pre-conceptional environmental factors that affect both the female and male germ lines.

One of the environmental hazards to which many Vietnamese have been exposed is Agent Orange, a highly toxic, dioxin-containing herbicide, which was used during the Vietnam War. In spite of its assumed toxicity, there has been considerable controversy about its long-term health effects [33,34], and it has not been confirmed if paternal exposure to Agent Orange can affect gene expression during spermatogenesis [35]. However, maternal exposure to dioxin and other components of pesticides have been associated with neurodevelopmental problems in the offspring [36,37]. Environmental exposures related to the Balkan wars include, for example, polychlorinated biphenyls (PCBs) [38] and depleted uranium [39]. Alternatively, psychosocial stress, which is common among refugee populations, might account for the finding. It has been suggested that maternal stress during pregnancy may affect fetal neurodevelopment and be a contributing factor to autism, possibly through epigenetic mechanisms [40].

The limitations of this study are as follows: The small number of immigrants from most countries did not allow a more detailed regional analysis. Only children born in Finland by first generation immigrants were included in the study. There was no information on the reason of immigration. Immigrant parents may not represent the general population of their country of origin for several reasons. The resources needed for employment-based migration in particular may only be available to a selected group of people. On the other hand, especially those refugees who are selected in the refugee quota often represent the most vulnerable parts of the population. Autistic traits in the family or other risk factors associated with autism in offspring may also influence the decision to migrate. Thus we cannot conclude if the increased risk of autism in certain immigrant populations could be explained by factors associated with their country of origin or by selective migration. There might also be residual confounding. Factors such as pre-migration living conditions, socioeconomic status and health behaviors other than smoking during pregnancy could not be included. Misclassification of diagnosis is a possibility, but this is not supported by the diagnostic validation study. However, it is possible that the rate of misdiagnosis would be higher if a child has a different cultural background than the clinician.

## Conclusion

The study showed that offspring of immigrant parents in Finland have an increased risk of childhood autism. Specifically the risk was increased in families in which the mother and/or the father was born in Vietnam or the former Yugoslavia, and in families in which the mother was born in the region of the former Soviet Union. Etiological factors specific to these regions or factors prevalent among these immigrants in Finland may play a role in the observed associations. Further studies in larger immigrant populations including first-generation immigrants and in the immigrants’ countries of origin are needed to confirm the findings. Studies that utilize biological markers of toxic or other exposures may facilitate identification of specific risk factors. Finally, it remains unclear whether migrant status affected the likelihood of being diagnosed with childhood autism. The functioning of the health care system and mental health services to detect childhood autism among migrants and ethnic minorities should be investigated.

## Competing interests

The authors declare that they have no competing interests.

## Authors’ contributions

VL contributed to the design of the study and interpretation of data and drafted the initial manuscript. SHY contributed to acquisition of data and was responsible for analysing it and critically reviewed the manuscript. KCP contributed to interpretation of data and critically reviewed and revised the manuscript. MG contributed to the design of the study and acquisition of data and critically reviewed and revised the manuscript. ASB contributed to the design of the study and interpretation of data and critically reviewed and revised the manuscript. AS contributed to the design of the study and interpretation of data and critically reviewed and revised the manuscript. All authors read and approved the final manuscript.

## Pre-publication history

The pre-publication history for this paper can be accessed here:

http://www.biomedcentral.com/1471-2431/13/171/prepub
